# The low-FODMAP diet and the gluten-free diet in the management of functional abdominal bloating and distension

**DOI:** 10.3389/fnut.2022.1007716

**Published:** 2022-11-08

**Authors:** Tommaso Pessarelli, Andrea Sorge, Luca Elli, Andrea Costantino

**Affiliations:** ^1^Gastroenterology and Endoscopy Unit, Fondazione IRCCS Ca’ Granda Ospedale Maggiore Policlinico, Milano, Italy; ^2^Department of Pathophysiology and Transplantation, University of Milan, Milan, Italy

**Keywords:** gluten-free diet (GFD), low-FODMAP diet, gut-brain interaction, functional bloating, abdominal distension, microbiota, functional diseases, diet

## Abstract

This review summarizes current knowledge on the role of low-FODMAP diet and gluten-free diet in functional abdominal bloating and distension, an emerging disorder of gut-brain interaction characterized by remarkable costs for healthcare systems and a significant impact on the patient’s quality of life. Ingested food plays a key role in the pathophysiology of disorders of gut-brain interaction as up to 84% of patients with irritable bowel syndrome (IBS) report food-triggered symptoms. Potential pathogenetic mechanisms of food-related symptoms in these patients are discussed, focusing on bloating and abdominal distension. These mechanisms provide the rationale for dietary treatment in patients with functional abdominal bloating and distension. The role of fermentable oligosaccharides, disaccharides, monosaccharides, and polyols (FODMAPs) and gluten in functional abdominal bloating and distension is examined. Current literature evaluating the efficacy of the low-FODMAP diet and the gluten-free diet in abdominal bloating and distension is analyzed. Available evidence originates mainly from studies on patients with IBS, since clinical studies on selected cohorts of patients with only functional abdominal bloating and distension have been missing to date. Promising evidence on the potential efficacy of the low-FODMAP diet in functional abdominal bloating and distension is provided by the reduction of the bloating observed in patients with IBS. Regarding the gluten-free diet, there is insufficient evidence to recommend it to reduce bloating and abdominal distension. In conclusion, this review asserts the need for a close collaboration with experts in nutrition to optimize the management of these patients and reduce the risks associated with elimination diets.

## Introduction

Disorders of gut-brain interaction (DGBI), previously known as functional gastrointestinal disorders, affect approximately 40% of the population worldwide (1). Accounting for more than one third of new patient referrals to tertiary hospitals, DGBI represent one of the leading causes of gastroenterological evaluation (2) and have relevant global health care costs (3, 4). Among gastrointestinal (GI) symptoms of patients with DBGI, abdominal bloating is one of the most frequent, affecting 3.5% of the general population (Female:Male = 2:1) (1) and accounting for approximately 23% of patients referred to a general gastroenterology clinic (2). The impact of chronic abdominal bloating and distension on quality of life is substantial since 75% of patients with bloating characterize their symptoms as moderate to severe (5).

According to the Rome IV criteria, functional abdominal bloating/distension (FABD) refers to recurrent bloating (a sensation of abdominal fullness, pressure, or trapped gas) and/or distension (a measurable increase in abdominal circumference) occurring on average at least 1 day/week, in patients with insufficient criteria for a diagnosis of other DGBI. Diagnostic criteria should be fulfilled for the last 3 months with symptom onset at least 6 months prior to diagnosis (6). Mild abdominal pain related to bloating and minor bowel movement abnormalities may be present as well, but sensations of bloating and/or abdominal distension predominate (6).

Up to 96% of patients with irritable bowel syndrome (IBS) report bloating and abdominal distension, with these symptoms frequently coexisting (7–9), since abdominal distension is reported by 50–60% of patients with bloating (10).

The pathogenesis for FABD is complex, often multifactorial in nature, and incompletely understood. Altered microbiota, abnormal GI motility, abdomino-phrenic dyssynergia, pelvic floor dysfunction, and visceral hypersensitivity are possible factors involved in the physiopathology of this condition (11). A full assessment of the principal secondary aetiologies of FABD (Table 1) must be performed when facing this condition (12).

**TABLE 1:** Differential diagnosis of abdominal bloating and distension.

**Dietary**
Excess of gas-producing foods
**Malabsorption**
Celiac disease
Lactose and fructose intolerance
Pancreatic insufficiency
**Functional gastrointestinal diseases**
Irritable bowel syndrome
Functional dyspepsia
Functional bloating/distension
Chronic idiopathic constipation
**Motility disorders**
Diabetes
Systemic sclerosis
Chronic intestinal pseudo-obstruction
Gastroparesis
Acute adynamic ileus
**Medications** (e.g., loperamide, opioids, and insoluble fiber supplements)
**Intestinal obstruction (mechanical)**
**Malignancy and cysts**
Gastrointestinal
Uro-genital
**Ascites**
**Infectious**
Small intestinal bacterial overgrowth
Giardiasis
**Psychological**
Anxiety (aerophagia)
**Physiological**
Pregnancy
Adiposity

Adapted from Abraczinskas (12).

The quality of ingested food plays a key role in the pathophysiology of DGBI and patients’ perception of symptoms (13). Nevertheless, there are no dietary guidelines for FABD due to the lack of high-quality studies on this specific condition. Currently, the most widely adopted dietary approaches for FABD are a low fermentable oligosaccharides, disaccharides, monosaccharides, and polyols (FODMAP) diet and the gluten-free diet (GFD). With regards to these exclusion diets the evidence supporting them is limited, especially for the GFD.

The aim of this review is to summarize the most relevant and recent evidence on the effects of GFD and the low-FODMAP diet (LFD) in the management of FABD, therefore providing an updated guide for clinicians in real-life daily practice to optimize the dietary management of these patients.

### Role of diet in disorders of gut-brain interaction

In recent years, clinical studies evaluating the relationship between dietary interventions and functional GI disorders have increased noticeably. As showed by Böhn et al. up to 84% of IBS patients and a large proportion of subjects with DGBI refer to food-triggered symptoms (13, 14). Several pathophysiological mechanisms have already been proposed to explain the impact of food on the onset of GI symptoms. One of these involves an alteration of GI mechanoreceptors, chemoreceptors, and thermoreceptors responsible for nutrients sensing (15, 16). Another possible underlining mechanism may be a defective processing of nutrients, as some studies suggested a potential role of fructose (17) or lactose intolerance (18) and of lactase-isomaltase polymorphism (19) in patients with DGBI. Food-triggered symptoms might also derive from the hypersensitivity to the distension of the bowel lumen, worsened by ingestion of low-absorbable high-osmolarity aliments (e.g., FODMAPs) (20). Aberrant gut microbiota is another etiologic factor often involved since it may increase FODMAP fermentation leading to lumen distension, production of neuroactive mediators with nociceptive signaling effect and increase of visceral hypersensitivity (21, 22).

According to another hypothesis, an immune-allergic reaction might lead to food-triggered symptoms in DGBI (23). According to this possible mechanism, increased GI permeability and the consequent passage of antigens through the tight junctions might induce a mast-cell mediated reaction leading to symptoms (24, 25).

The available evidence, despite mostly deriving from studies on food-related symptoms in patients with IBS, supports the key role of dietary interventions in the management of patients with DGBI, thus including FABD.

### Dietary approaches in the management of functional abdominal bloating/distension

Not all patients with FABD are optimal candidates to dietary prescriptions. In fact, dietary interventions may be mostly effective in patients with meal-related GI symptoms, especially in those who are motivated to introduce the necessary changes in their daily diet. Before dietary prescription, a full nutritional assessment is recommended since restrictive diets should be avoided in patients at risk for malnutrition (26). Moreover, a careful evaluation of dietary history is key since elimination diets may exacerbate psychiatric eating disorders (26). Therefore, a close collaboration with dietitians and nutrition specialists should be offered to optimize the management of these patients. Despite its importance, access to these professional figures can be limited in some healthcare systems.

Due to the lack of clear evidence on symptoms improvement with specific diets in FABD, current dietary prescriptions are mainly based on an empirical approach. A possible approach for FABD patients is the elimination of gas-producing foods (e.g., onions, legumes, cauliflower, celery, bananas, apricots, prunes, broccoli, and wheat bran) and the recommendation of healthy eating habits [e.g., regular meals; restriction of alcohol, coffee, and spicy foods (27, 28)]. In addition, patients are often advised to eliminate foods that trigger their GI symptoms (29). Apart from these approaches, the two most frequently adopted dietary prescriptions are the LFD and the GFD (11).

#### The low-fermentable oligosaccharides, disaccharides, monosaccharides, and polyol diet in the management of functional abdominal bloating/distension

##### Pathophysiology

**FIGURE 1:**
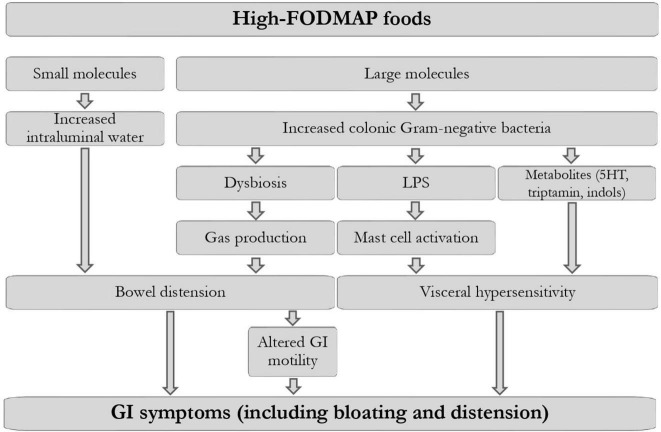
Proposed pathogenetic mechanisms leading to gastrointestinal symptoms, including bloating, after FODMAPs ingestion in predisposed individuals. Modified from Singh et al. (47) and Van den Houte et al. (16). LPS, lipopolysaccharide; 5HT, 5-hydroxytryptamine; GI, gastrointestinal.

Fermentable oligosaccharides, disaccharides, monosaccharides, and polyols include lactose, fructose in excess of glucose, sugar polyols (sorbitol and mannitol), fructans, and galacto-oligosaccharides (30). The role of FODMAPs in pathogenesis of functional GI symptoms has been studied mainly in IBS patients. However, due to the significant symptomatic and pathogenetic overlap between FABD and IBS, is conceivable that the same pathophysiological mechanisms are involved (Figure 1). A clear mechanism involves bowel distension; small molecules like fructose, mannitol, sorbitol, and lactulose all exert a direct osmotic force, which increases intraluminal water (31). This has been demonstrated in studies in which a diet high in sucrose, polyols and fermentable carbohydrates significantly increased total effluent wet weight from ileostomy (32, 33). It was then confirmed in studies that measured small bowel water content through magnetic resonance imaging (MRI), which revealed a significant increase in intestinal water volume after ingestion of 17.5 g mannitol solutions or 40 g of fructose (34, 35). On the other hand, longer molecules or fermentable carbohydrates non-digested and/or non-absorbed in the small intestine distend the colon through hydrogen and methane produced by microbial fermentation (35). Interestingly, a recent study revealed that, after FODMAPs ingestion, breath hydrogen, and colonic volume kinetics measured by MRI were equally increased in IBS patients and healthy individuals (36).

Another study based on computed tomography imaging showed that luminal gas increases only in 25% of patients with functional GI disorders during a spontaneous episode of abdominal distension or following consumption of a “high-flatulence” diet, thus questioning the role of luminal distension in the genesis of bloating and abdominal distension (37). Hence, visceral hypersensitivity to distension rather than excess of colonic gas may be largely responsible for food-related symptoms (38).

Another possible mechanism underlying FODMAP-related symptoms in FABD is aberrant intestinal motility since bowel distension can lead to an acceleration of bowel transit. Madsen et al. showed that the small intestinal transit time is decreased following ingestion of a 30 g fructose-sorbitol mixture in healthy individuals (39).

A high-FODMAP diet may also lead to dysbiosis and visceral hypersensitivity, pathogenetic factors strictly related with FABD. Rodent studies suggest that FODMAPs may cause dysbiosis and an increase in gram-negative bacteria, and consequently the luminal lipopolysaccharide, which can activate mast-cells (22, 40). Subsequently, tryptase, histamine, and prostaglandin E2 released by mast-cells can increase intestinal permeability and cause visceral sensitivity. Furthermore, a FODMAP-rich meal has been shown to increase hydrogen production and induce GI symptoms in IBS, in association with metabolome and microbiota alterations (41). In fact, an imbalance in the intestinal microbiota species may lead to aberrant by-products (e.g., methane and hydrogen) showing that the relationship between microbiota and FODMAP-containing food in the genesis of bloating and abdominal distension could be bidirectional (42).

##### Characteristics of the low-fermentable oligosaccharides, disaccharides, monosaccharides, and polyol diet

Several studies reported the average FODMAP content of the majority of foods (43–45). Consequently, the criteria for classifying a food as low in FODMAPs were defined according to specific cut-off values for each FODMAP element including fructose in excess of glucose, oligosaccharides (fructans plus galacto-oligosaccharides), polyols (sorbitol and mannitol), and lactose (46). More specifically a low-FODMAP food must contain:

– less than 0.3 g per serve (g/serve) or less than 0.2 g/serve of oligosaccharides (for core grain products, legumes, nuts and seeds or for vegetables, fruits, and all other products, respectively)
– less than 0.4 g/serve of total polyols
– less than 0.4 g/serve of excess fructose
– less than 1 g/serve of lactose.

The LFD consists of three phases. In the first phase, ideally lasting 2–8 weeks, the aim is to restrict the intake of high-FODMAP foods, substituting them with suitable low-FODMAP aliments of the same food group (47). In the absence of clinical benefit after this period, a trial with an alternative treatment is advisable. In the second phase, a rechallenge with single high-FODMAPs-containing foods, gradually reintroduced by patient’s nutritional needs and preferences, is performed every 2–3 days to enable identification of specific food triggers and reintroduction of tolerated foods into the diet (48). In the third phase a personalized maintenance diet should be defined, by avoidance of foods causing severe symptoms and reintroduction of well-tolerated, FODMAPs-containing foods. Notably, adherence to the second and third phases might be unsatisfying in the absence of support by a dietitian. New technologies, such as mobile applications (49) or telemedicine (50) may be helpful in increasing adherence to dietician recommendations and improving outcomes.

In the last decade, some randomized controlled trials (RCTs) and meta-analyses showed that the first phase of the diet is efficacious for global IBS symptoms (51–53). Thus, both the National Institute for Health and Care Excellence guidelines and the American College of Gastroenterology guidelines recommend the LFD for the management of IBS (27, 54), with both guidelines underlining the necessity of a trained GI dietician to monitor patients during the diet. In fact, the dietician’s work is key to tailor a balanced, varied and minimally restrictive diet.

##### Efficacy

Although studies evaluating the effects of LFD on selected cohorts of patients with FABD are scant, a low-FODMAPs diet may have a beneficial effect in FABD through the proposed pathogenetic mechanisms (see Paragraph 3.1.1).

Indirect evidence on the potential efficacy of LFD in FABD is provided by the reduction of bloating in patients with IBS. We included in this narrative review all meta-analysis assessing the effects of LFD on bloating in patients with IBS. Clinical trials contained in the meta-analysis were not discussed singularly to avoid redundancy. We also summarized some recent RCTs studying the efficacy of LFD on bloating and abdominal distention in other conditions than IBS.

The meta-analysis by Marsh et al. included six RCTs in which bloating (among other GI symptoms) was assessed at baseline and after 3–6 weeks in a total of 182 patients who underwent restriction of high-FODMAP foods and in 172 patients of control groups who continued their standard diet (55). This study showed a significant reduction of bloating, assessed through the IBS severity scoring system (IBS-SSS), in patients following the LFD.

In a more recent meta-analysis, Black et al. selected 12 RCTs that studied the effect of an LFD on GI symptoms, including abdominal bloating and distension in adult patients with IBS of any subtype (56). Trials that were included in this meta-analysis compared the effects of 3–6 weeks of LFD with an alternative intervention that could consist of any of habitual diet, sham dietary advice, a high-FODMAP diet or alternative dietary advice. The LFD ranked first in bloating improvement compared to all other dietary interventions, with statistical significance if compared with British Dietetic Association dietary advice. Another meta-analysis including three RCTs comparing 4 weeks LFD versus traditional IBS diet, three RCTs comparing LFD and high-FODMAP diet (11 days to 3 weeks of intervention) and six cohort studies comparing IBS symptoms (including bloating) at basal level and after 3 weeks to 15 months of LFD revealed a significant improvement of bloating in the low-FODMAP groups (57).

Some RCTs investigated the effects of LFD in other conditions than IBS. In a recent RCT, 19 patients with ulcerative colitis in remission and concomitant IBS underwent an 8-week randomized FODMAP elimination with double-blinded, crossover provocations of FODMAP and placebo (58). Eliminating FODMAPs-containing foods for 2 weeks resulted in a 56% reduction of bloating, assessed through Visual Analog Scale (VAS). Eliminating FODMAPs-containing foods for 2 weeks resulted in a 56% reduction of bloating, assessed through the visual analog scale. After the subsequent provocations, bloating scores returned to baseline levels in both FODMAPs and placebo groups, perhaps due to the known nocebo effect in patients with DGBI (59, 60).

In another RCT, patients with a quiescent inflammatory bowel disease and functional GI symptoms responsive to the LFD were allocated either to a 3-day fructan challenge or a 3-day glucose challenge. There was greater severity of bloating on the final day of the fructan challenge compared with glucose (61).

In another RCT, 105 patients with functional dyspepsia were randomized in the LFD group and in a traditional dietary advice group. This study revealed that the subgroup of patients with associated bloating had significantly better symptomatic response with LFD and on multivariate analysis, one of the two factors predicting response to LFD was bloating (62).

#### The gluten-free diet in the management of functional abdominal bloating/distension

##### Pathophysiology

Celiac Disease (CD) and Wheat Allergy (WA) are classically considered as the two main gluten-related diseases, accounting for 1% prevalence in general population and pediatric population, respectively (63, 64). CD diagnosis requires clinical, serological and histological parameters while diagnosis of WA requires typical allergic symptoms associated with wheat assumption and the presence of specific IgE (65, 66).

In the last decade, a considerable rise in self-reported wheat sensitivity and avoidance of gluten in absence of a diagnosis of CD has been described (67) and it has been suggested that gluten might also play a role in DGBI, as some patients with IBS report GI and extra-intestinal symptoms after the ingestion of gluten (68). Consequently, the gluten-free industry grew exponentially, reaching estimated yearly retail sales of USD 24 billion (69). Large population studies suggested a prevalence of self-reported wheat sensitivity between 4 and 15% of the general population (67, 69–71).

The Salerno Experts’ Criteria defined non-celiac gluten sensitivity (NCGS) as a condition characterized by intestinal symptoms, the most frequent being bloating and abdominal pain, and extra-intestinal symptoms related to the ingestion of gluten-containing food in subjects where CD and WA had been excluded (72). A full diagnostic workup for NCGS should assess the clinical response to a GFD and then measure the effect of a gluten challenge (72).

A realistic estimate of non-celiac gluten/wheat sensitivity (NCGWS) world prevalence is lacking, mainly due to high rates of self-diagnosis and lack of standardized international diagnostic criteria or biomarkers (73), but it has been suggested that NCGWS might affect a large proportion of the population ranging from 0.6 to 13% (74, 75).

The clinical presentation of NCGWS may be indistinguishable from that of many DGBI (thus including FABD), leading some experts to suggest that NCGWS may represent a subgroup of IBS (wheat-sensitive IBS) (76, 77). Nevertheless, approximately 70% of patients considered to have NCGWS report bloating (11, 78), thus supporting the potential beneficial effect of the GFD in highly selected patients with FABD.

##### The gluten-free diet: Characteristics and open issues

A commonly accepted cut-off to define the gluten contamination potentially generating symptoms and/or histologic alterations in celiac patients is 10 mg of gluten, or 500 g of food containing 20 milligrams/kilogram of gluten. However, there is a tremendous degree of variability within this population, since some patients may have worsening histological changes with very low daily gluten exposure (79). Due to clinical overlap with other DGBI, a similar cut-off was not defined for NCGWS patients. The average daily gluten intake in a Western diet is thought to vary from 5 to 20 g/day, with wheat-containing bread being one of major sources of gluten (each slice of bread contains approximately 4 g of gluten) (80).

A strict adherence to the GFD is the only safe and recommended treatment for patients with CD, whereas for patients with NCGWS and wheat-sensitive IBS a clear benefit is far from being proven.

The GFD consists of the complete exclusion of wheat, rye, and barley from the diet. Although commercial oat products are frequently labeled as gluten-free, they might be are often contaminated (81). Besides gluten, several components of these cereals are potentially involved in the pathogenesis of GI diseases and symptoms (82). As an example, amylase–trypsin inhibitors can cause wheat allergies and FODMAPs can induce symptoms of IBS (83).

GFD is associated with potential drawbacks. When eliminating gluten, patients may reduce variety, balance, and nutritive value of their diet, as gluten-free products are often low in fiber, vitamins, and other micronutrients (e.g., calcium, iron, and magnesium). In any case, common gluten-free products are often enriched to reduce the risk of nutritional imbalances. Higher levels of lipids, trans fat, protein, and salt were reported in studies evaluating the nutritional composition of processed gluten-free products (84–87). Notably, poor nutrition is not the only potential issue of the GFD. Although the overall effect of the GFD on cardiovascular risk is unclear, many studies showed that it may increase total cholesterol, high-density lipoprotein, fasting glycemia, and body mass index (88–91). Thus, to ensure the safety and efficacy of the GFD, the indication should be evaluated by a specialized gastroenterologist and a dietary planning guidance from a nutritionist or dietitian should be provided to assess the fulfillment of all nutritional needs and prevent deficiency of key nutrients and malnutrition (68, 92). To avoid the risk of malnutrition in patients with gluten-related disorders, a combination of the Mediterranean diet and the GFD has been proposed as an alternative to reach a healthy gluten-free state (93). In addition, the GFD may have a considerable psychological impact and interfere with social life, also due to low availability, high costs, and food labeling of gluten-free products, which may also limit adherence to the diet (94).

##### Efficacy

Considering the clinical overlap between NCGWS and DGBI (including FABD), there may be a rationale for prescribing the GFD to highly selected patients with FABD, especially to those reporting gluten-related symptoms. However, as for the LFD, scientific data about the effects of GFD in patients with FABD are lacking and the few results on the potential efficacy of GFD in bloating and abdominal distension come from studies on patients with IBS. We analyzed the only two RCTs and the only meta-analysis evaluating the effects of GFD on bloating in IBS. We also reported the only clinical trial assessing the effects of a gluten challenge on bloating in NCGS and the only meta-analysis comparing LFD, GFD and balanced traditional diet on bloating in IBS.

A RCT of 34 IBS patients symptomatically controlled on a GFD showed a significant worsening of symptoms, including bloating, after ingestion of gluten for up to 6 weeks compared to the placebo control group (95).

In another double-blind randomized placebo-controlled trial, 72 IBS patients followed a GFD for up to 6 weeks and then either received powdered gluten or placebo. In the gluten-containing group all symptoms, including bloating, significantly improved after 6 weeks of GFD and significantly increased after 1 week of gluten reintroduction (96).

A recent meta-analysis including the two previously reported RCTs showed results that were not statistically significant due to the low number of studies and patients, and the high heterogeneity between individual trial results. This suggests that there is insufficient evidence to recommend a GFD to reduce bloating and abdominal distension (53).

In another RCT, 61 adults with a diagnosis of NCGS were assigned to groups given either low quantities of gluten or a placebo for 1 week, with subsequent crossover. This study revealed that intake of gluten significantly increased the severity of abdominal bloating (97).

A recent clinical trial investigated the effects of 4 weeks of LFD, GFD, and a balanced traditional diet (12 total weeks of diet) on 42 IBS patients, showing a beneficial effect of all three diets on bloating (98). The comparison of the three diets revealed a superiority of the LFD over the others in decreasing abdominal bloating. However, a limitation of this study is that LFD was administered as a first diet in all patients, thus potentially contributing to this result.

## Conclusion

Functional abdominal bloating/distension is a highly prevalent clinical entity that, due to the clinical overlap with IBS and other DGBI, is frequently underdiagnosed. Hence, studies on selected cohorts of patients with this condition are lacking. Dietary treatment is currently a cornerstone of the management of several DGBI, including FABD.

Currently, two of the most frequently prescribed dietary regimens in FABD are the LFD and the GFD. Although specific studies on patients with FABD are needed to investigate the role of different dietary regimens, current evidence may justify the prescription of the LFD in these patients, while the recommendation of the GFD is not currently supported by clear evidence.

In conclusion, although studies in patients with IBS and bloating revealed promising results, especially for the LFD, interventional studies on patients affected by FABD are needed to increase our knowledge on the efficacy of dietary interventions in this condition.

## Author contributions

TP, AS, and AC: conceptualization. TP and AS: writing of the original draft. TP, AS, LE, and AC: critical revision of the manuscript and editing. All authors contributed to the article and approved the submitted version.
